# Identification of MAPK Genes in *Phaseolus vulgaris* and Analysis of Their Expression Patterns in Response to Anthracnose

**DOI:** 10.3390/ijms252313101

**Published:** 2024-12-05

**Authors:** Huiling Liu, Da Wang, Zhenyu Wang, Tong Zhao, Jingying Zhang, Yan Wang, Hongyu Qiao, Yuzhu Han

**Affiliations:** Modern Vegetable Industry Technology and Germplasm Resource Innovation Team, Northeast Asia Special Germplasm Resource Conservation and Innovation Center Vegetable Breeding Technology Innovation Team, College of Horticulture, Jilin Agricultural University, Changchun 130118, China; liuhuiling@mails.jlau.edu.cn (H.L.); 2114100408@mails.jlau.edu.cn (D.W.); 20221235@mails.jlau.edu.cn (Z.W.); zhaotong@mails.jlau.edu.cn (T.Z.); zhangjingying@jlau.edu.cn (J.Z.); wangyan@jlau.edu.cn (Y.W.)

**Keywords:** kidney bean (*Phaseolus vulgaris* L.), MAPK cascade, genome-wide identification, gene expression analysis, phylogenetic analysis

## Abstract

The oil bean is a high-quality, economically valuable variety of kidney bean (*Phaseolus vulgaris* L.) that is widely cultivated in Northeast China. However, the prevalence of anthracnose, caused by a combination of factors, including continuous cropping over many years, has led to significant declines in both yield and quality. The mitogen-activated protein kinase (MAPK) cascade is a highly conserved plant cell signaling pathway that plays a pivotal role in plant growth and development, as well as responses to biotic stress. However, its role in the response of *P. vulgaris* to anthracnose infection has not previously been reported. We identified and characterized thirteen *MAPK* genes (*PvMAPK01–PvMAPK13*) in the *P. vulgaris* genome. These genes were found on eight of the eleven chromosomes of *P. vulgaris*, and phylogenetic analyses classified them into four previously established subgroups (A–D). Analysis of the *cis*-acting elements in their promoter regions revealed the presence of multiple elements associated with light, hormone regulation, stress responses, and growth and development. An analysis of intraspecific collinearity revealed that whole-genome and/or segmental duplication, rather than tandem duplication, has been the primary driver of *PvMAPK* family expansion in *P. vulgaris*. Transcriptome data revealed that the *PvMAPKs* differed in their tissue-specific expression patterns, with *PvMAPK05* showing particularly high expression in stems and stem tips and *PvMAPK07* and *PvMAPK11* showing relatively low expression across all tissues. In general, expression of the *PvMAPKs* was higher in stems, stem tips, and pods than in other tissues and organs, suggesting that they may be particularly important for regulating stem and pod development. Analysis of the expression of *PvMAPKs* in field-grown plants infected or uninfected with anthracnose revealed that the relative expression levels of *PvMAPK05*, *PvMAPK07*, *PvMAPK09*, and *PvMAPK11* exhibited particularly significant changes in response to anthracnose infection across different varieties, suggesting their potential involvement in the anthracnose response of Phaseolus vulgaris. This study reports the fundamental characteristics of the thirteen *MAPK* genes in *P. vulgaris*, documents their expression patterns in diverse tissues, and offers preliminary insights into their responses to anthracnose infection, establishing a foundation for subsequent functional validation.

## 1. Introduction

The oil bean (*Phaseolus vulgaris* L.) is a high-quality kidney bean variety that is indigenous to the northeast region of China [1]. It is one of the region’s most distinctive agricultural products, offering a combination of high nutritional value and significant economic benefit. However, owing to the practice of continuous cropping or seed carrying, pathogens have become increasingly prevalent, and disease incidence has worsened from year to year. Among these pathogens, anthracnose is of particular concern; it affects the leaves, stems, and pods of the oil bean, resulting in significant yield and commercial losses, with estimates reaching 30–90% [2]. Research has shown that *Colletotrichum lindemuthianum* is the primary causal agent of anthracnose in *P. vulgaris* [3], and pesticide spraying is the primary method for anthracnose control in commercial production. However, excessive reliance on pesticide spraying can lead to diminishing returns and can cause soil contamination in agricultural fields. Exploring ways to harness or manipulate the immune system of *P. vulgaris* for anthracnose prevention and control will therefore be important for advancing the ‘green defense revolution’ and the ‘agriculture and nature coordinated development’ major strategy [4].

Plants are typically exposed to multiple stresses during their growth and development [5], and they have developed a variety of molecular mechanisms to adapt to these stresses over the course of long-term evolution. Stress-response mechanisms involve the transduction of external environmental signals into molecular and physiological responses, a process that is regulated by signal transduction pathways. Plant signal transduction pathways are numerous and diverse. One such pathway is the mitogen-activated protein kinase (MAPK) cascade, a widespread and highly conserved eukaryotic signaling module that converts the signals generated by cell membrane receptors into cellular responses, thereby regulating plant growth and development. A typical MAPK cascade involves a series of phosphorylations mediated by three types of kinase: (1) mitogen-activated protein kinase (MAPK), (2) MAPK kinase (MAPKK), and (3) MAPKK kinase (MAPKKK) [6].

Upon perception of an external environmental stimulus, a plant cell membrane receptor transduces this signal to the MAPK cascade pathway. The first element of the cascade to be activated by phosphorylation is MAPKKK. As the signal is transduced in a sequential manner, MAPKK and MAPK are in turn activated by phosphorylation. Finally, the activated MAPK regulates its downstream response factors (typically transcription factors or other types of kinases), which amplify the extracellular signal and initiate cellular responses. It has recently been demonstrated that MAPKKKs can be activated not only by membrane receptors but also by MAPKKKKs, whose function remains poorly understood.

The mitogen-activated protein kinases (MAPKs) are located at the end of the MAPK cascade and act as hubs connecting the upstream and downstream substrates of the cascade pathway, resulting in increased complexity and sequence diversity. MAPKs have 11 highly conserved structural subdomains, and the activation loop between domains VII and VIII contains a TEY or TDY phosphorylation site that enables MAPK activation and interaction with upstream MAPKK activators [7,8]. Plant MAPKs can be classified into four groups, A–D. Members of groups A, B, and C have a TEY motif at the phosphorylation site, whereas members of group D have a TDY motif; the latter are found only in plants. The MAPK gene family has been systematically characterized in a number of plant species, including Arabidopsis (*Arabidopsis thaliana*, 20 genes) [9], *Brachypodium distachyon* (16 genes) [10], maize (*Zea mays*, 19 genes) [11], barley (*Hordeum vulgare*, 20 genes) [12], wheat (*Triticum aestivum*, 54 genes) [13], and rice (*Oryza sativa*, 17 genes) [14].

The MAPK cascade plays a key role in plant responses to biotic and abiotic stresses, such as pathogen infections [15]. Through phosphorylation, the MAPK cascade can regulate intracellular protein substrates and initiate cell signaling pathways, thereby regulating the expression of many defense genes and enhancing plant immunity [16]. For example, three OsMAPK genes are involved in rice blast resistance. The VQ motif proteins OsVQ14 and OsVQ32 act as substrates of the OsMPKK6–OsMPK4 cascade, promoting rice resistance to *C. oryzae* [17].

*OsMAPK20-5* has been identified as a positive regulator, and its silencing increased the susceptibility of transgenic plants to rice blast. By contrast, *OsMAPK5* and *OsMPK15* have been characterized as negative regulators, and suppression of *OsMAPK5* expression significantly increased blast resistance. Likewise, Wang et al. [18] demonstrated that the cotton *MKK4*–*MPK20*–*WRKY40* cascade plays a pivotal role in resistance to *Fusarium acnes*. Song Q-M [19] transiently expressed watermelon *ClMPK7* in tobacco and demonstrated its ability to induce the accumulation of reactive oxygen species and the expression of defense genes, thereby enhancing resistance to *Botrytis cinerea*. Similar findings have been found in other plants, such as in Arabidopsis thaliana, where AtMPKK1/AtMPKK2 or AtMPKK6 activate AtMPK4, which in turn phosphorylates AtVQ21 to promote defense responses, suggesting the importance and conserved nature of MAPK signaling pathways in plant immunity [20].

The aforementioned studies demonstrate that MAPK proteins can play a significant role in plant responses to biotic stress. Nevertheless, this phenomenon has been reported for only a few species, including *Arabidopsis*, rice, and several other crop plants, and little is known about the response of MAPK proteins to anthracnose in *P. vulgaris*. We therefore used MAPK sequences from *Arabidopsis* and rice to identify MAPK homologs in *P. vulgaris* and performed bioinformatics analyses to characterize their physicochemical properties, intra- and interspecific collinearity, and evolutionary relationships. We examined expression of the *PvMAPKs* in seven tissues of *P. vulgaris* using transcriptome sequencing and performed qRT-PCR to document changes in their expression in response to anthracnose infection. The results provide a foundation for further functional studies of the *PvMAPKs* and their use in preventing anthracnose-related damage in *Phaseolus*.

## 2. Results

### 2.1. Identification of PvMAPK Genes and Characterization of PvMAPK Proteins in P. vulgaris

**Figure 1:**
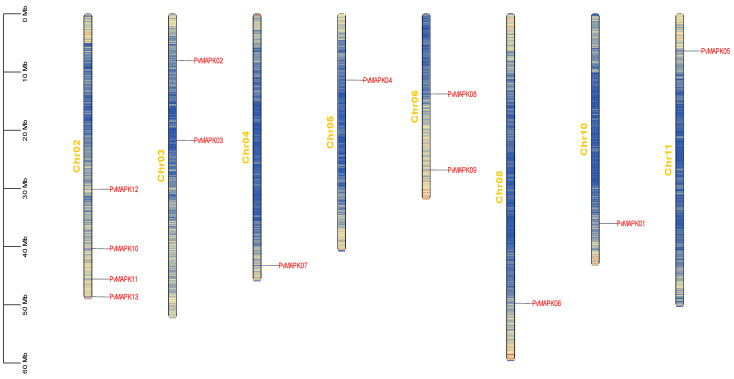
Locations of *PvMAPK* genes on eight of the eleven *P. vulgaris* chromosomes.

We identified 13 members of the MAPK gene family in the *P. vulgaris* genome (Table 1) and designated them *PvMAPK01*–*PvMAPK13*. The *PvMAPKs* were located on eight of the eleven *P. vulgaris* chromosomes: four on chromosome 2, two each on chromosomes 3 and 6, and one each on chromosomes 4, 5, 8, 10, and 11 (Figure 1).

The PvMAPK proteins ranged from 372 to 614 amino acids in length, with molecular weights of 42.48–69.98 kDa and isoelectric points of 5.65–9.21; all contained TEY or TDY phosphorylation sites (Table 1). Subcellular localization predictions suggested that 11 PvMAPKs were located in the cytoplasm, with only PvMAPK04 and PvMAPK06 located in the nucleus; none of the PvMAPKs were predicted to contain a signal peptide.

### 2.2. Phylogenetic and Collinearity Analyses of the P. vulgaris MAPK Gene Family

**Figure 2:**
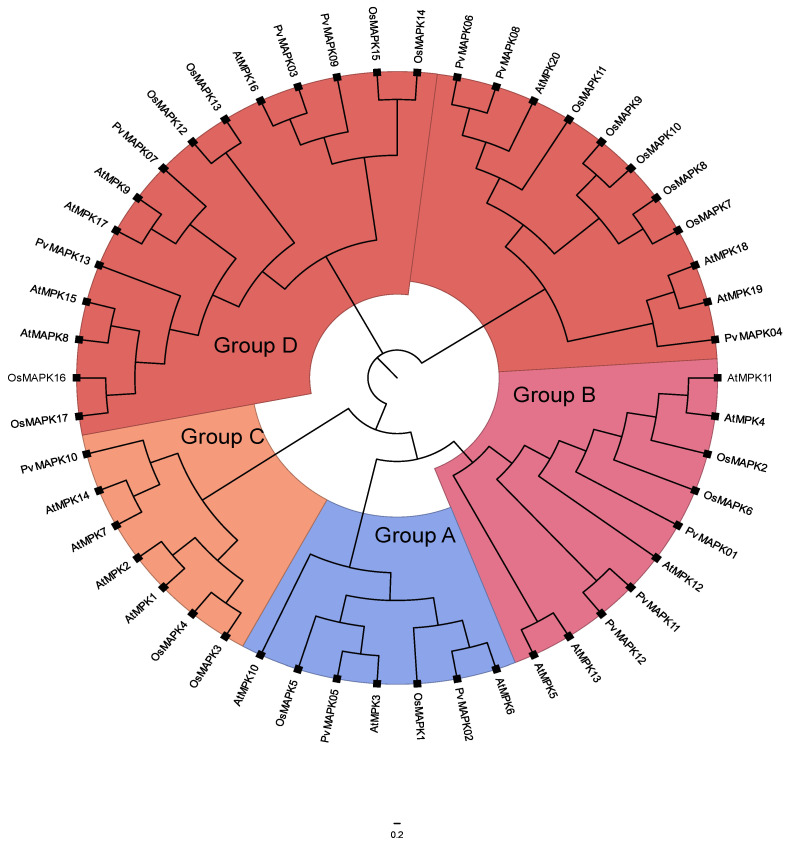
Phylogenetic evolution tree of the MAPK gene family in *Arabidopsis thaliana*, *Oryza sativa*, and *Phaseolus vulgaris*. Pv: *Phaseolus vulgaris*, At: *Arabidopsis thaliana*, Os: *Oryza sativa*.

To investigate the evolutionary relationships among the *MAPK* genes, we constructed a phylogenetic tree using the amino acid sequences of 20 AtMAPKs from *Arabidopsis*, 13 PvMAPKs from *P. vulgaris*, and 17 OsMAPKs from rice (Figure 2). The results revealed that the 13 PvMAPKs could be classified into the known MAPK groups: groups A–C with the TEY motif and group D with the TDY motif. Seven of the 13 PvMAPKs belonged to group D, which also contained the largest number of *Arabidopsis* and rice members. Among the remaining PvMAPKs, two belonged to Group A (PvMAPK02 and PvMAPK05), three to group B (PvMAPK01, PvMAPK11, and PvMAPK12), and one to Group C (PvMAPK10).

**Figure 3:**
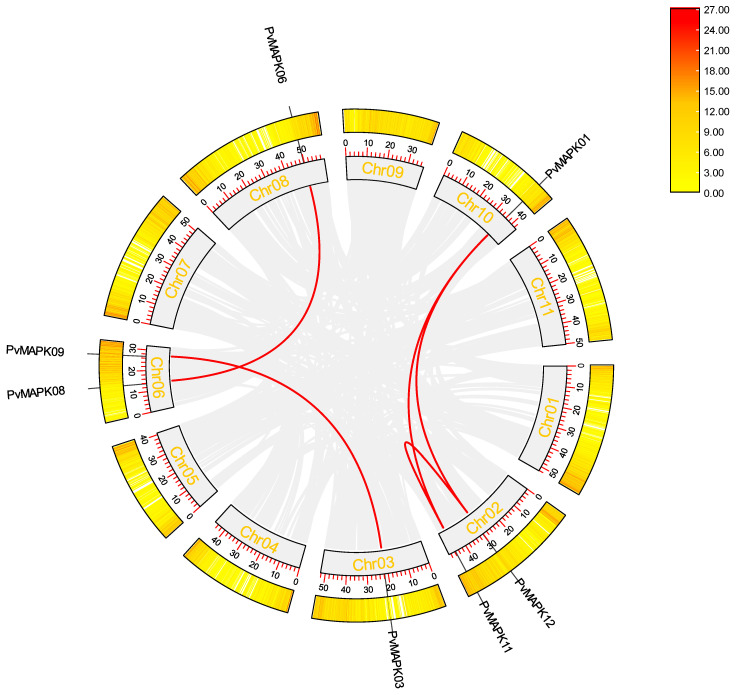
Identification of collinear *PvMAPK* gene pairs on *P. vulgaris* chromosomes. The outermost circle shows gene density, and the red lines connect syntenic *PvMAPK* gene pairs.

Analysis of intragenomic collinearity within the *P. vulgaris* genome (Figure 3) revealed the presence of five syntenic *PvMAPK* gene pairs but no apparent tandem duplications. Four of the syntenic pairs were located on different chromosomes (*PvMAPK06*/*PvMAPK08*, *PvMAPK03*/*PvMAPK09*, *PvMAPK01*/*PvMAPK11*, and *PvMAPK01*/*PvMAPK12*), whereas *PvMAPK11* and *PvMAPK12* were both located on chromosome 2. These results suggest that whole-genome and segmental duplication, rather than tandem duplication, has been the primary driver of PvMAPK family expansion in *P. vulgaris*. Ka/Ks calculations for these five collinear gene pairs (Table 2) revealed Ka/Ks ratios less than 1, indicating that the *PvMAPK* genes have been subjected to purifying selection during their evolution.

**Figure 4:**
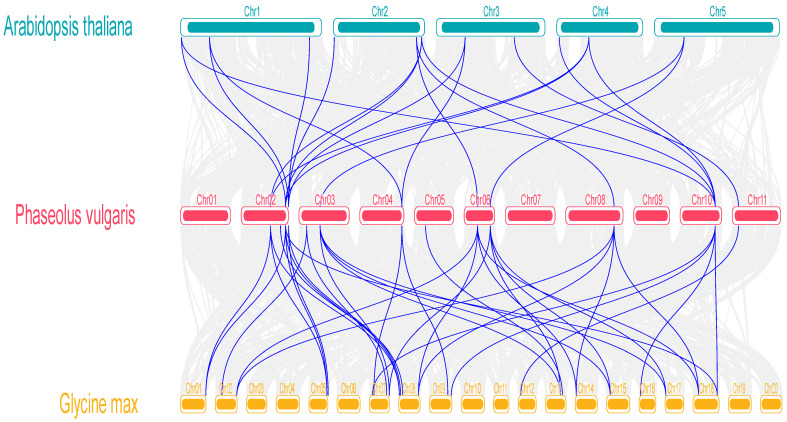
Intergenomic collinearity analysis of *P. vulgaris* with *A. thaliana* (**top**) and *G. max* (**bottom**). Blue lines connect syntenic pairs of *MAPK* genes between species.

We also examined collinear relationships among the *PvMAPKs* and *MAPK* genes from *Arabidopsis* and soybean (*Glycine max*). We identified 20 collinear *MAPK* gene pairs between *P. vulgaris* and *Arabidopsis* and 36 collinear gene pairs between *P. vulgaris* and soybean (Figure 4). The relatively close evolutionary relationships among these collinear gene pairs may provide insight into the functions of the *PvMAPKs*, although such predicted functions will require experimental verification.

### 2.3. Conserved Domains, Conserved Motifs, and Gene Structures of the PvMAPKs

**Figure 5:**
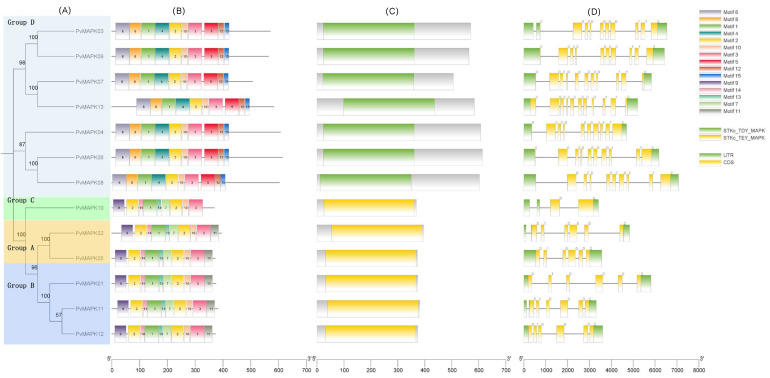
Conserved protein motifs, conserved protein domains, and gene structures of PvMAPK family members in *P. vulgaris*. (**A**) Neighbor-joining phylogenetic tree of the *PvMAPKs*. (**B**) Conserved motifs identified in the PvMAPK proteins using MEME tools. Sequence information for each motif is presented in Figure 6. (**C**) Conserved domains of the PvMAPK proteins STKc_TDY_MAPK (cd07859) and STKc_TEY_MAPK (cd07858). (**D**) Exon–intron structures of the *PvMAPK* genes. Green, untranslated region; yellow, coding sequence; gray line, intron.

**Figure 6:**
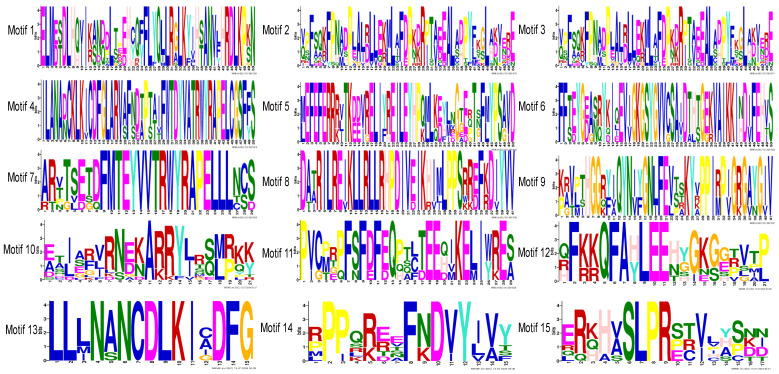
Sequence logos of 15 conserved motifs identified in the *P. vulgaris PvMAPK* proteins using MEME tools.

Conserved domains were identified in the 13 *PvMAPKs* using NCBI CD-search, and all were found to contain a pro-mitogen-activated protein kinase structural domain, either STKc_TDY_MAPK (cd07859) or STKc_TEY_MAPK (cd07858) (Figure 5). Conserved motifs were also identified in the *PvMAPKs* using MEME tools. The number and order of motifs were similar across the *PvMAPKs*, and all contained Motifs 1, 2, 3, and 10. Motif 4 included the characteristic TDY motif and was therefore present only in group D. Motif 7 included the characteristic TEY motif and was therefore found in groups A–C. The group A–C proteins also contained a distinctive N-terminal motif, Motif 9, whereas group D members contained a different N-terminal motif, Motif 6. At the C terminus, group A and B members contained motif 11, whereas group D members contained motif 15; all group D members also had long 3′ untranslated regions. Sequence logos of all the conserved motifs are shown in Figure 6.

The exon–intron structures of the 13 *PvMAPK* genes are shown in Figure 5. Exon numbers were similar among *PvMAPKs* from the same group: 9–11 for group D, 6 for groups A and B, and 2 for group C. The structures, and likely the functions, of *PvMAPKs* from different groups have clearly undergone differentiation over the course of evolution.

### 2.4. Identification of Cis-Acting Elements in the PvMAPK Promoters

**Figure 7:**
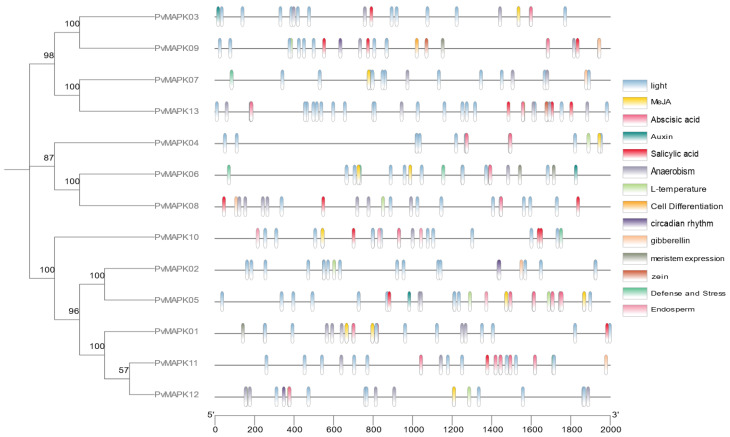
*cis*-elements identified in the *PvMAPK* promoters (2000 bp upstream of the start codon).

**Figure 8:**
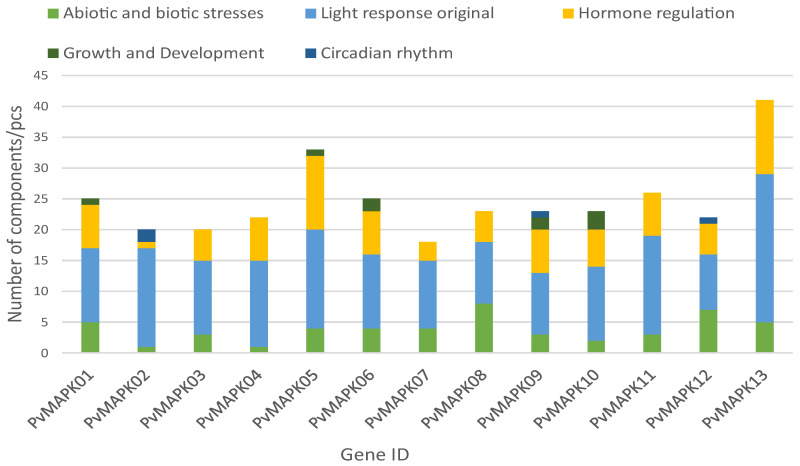
Numbers of promoter *cis*-elements from different classes in the 2000-bp upstream regions of the *PvMAPK* genes.

To gain insight into the potential functions of the *PvMAPKs*, we identified *cis*-acting elements in their 2000-bp upstream promoter regions (Figure 7). In addition to the conserved CAAT and TATA boxes, the *PvMAPK* promoters also contained 14 *cis*-acting elements, which we classified into five broad categories: light, stress, hormone regulation, growth/development, and circadian rhythm (Figure 8). Light-responsive elements were present in all *PvMAPK* genes, and they were the most abundant elements in all *PvMAPK* promoters. The second category, hormone regulation, included elements involved in responses to abscisic acid (present in 11 *PvMAPK* promoters), jasmonic acid methyl ester (9 promoters), auxin (3 promoters), gibberellin (5 promoters), and salicylic acid (8 promoters), as well as elements related to the metabolism of zein and similar prolamin proteins (2 promoters). In the stress category were elements related to hypoxia (11 promoters), low temperature (6 promoters) and defense and stress (4 promoters). Among the elements involved in plant growth and development were elements related to meristem expression (three promoters), endosperm expression (two promoters), and cell differentiation (*PvMAPK09* only). Finally, *cis*-acting elements associated with the control of circadian rhythm were present in three of the gene promoters (*PvMAPK02*, *PvMAPK09*, and *PvMAPK12*).

### 2.5. Expression of the PvMAPKs in Different P. vulgaris Tissues

**Figure 9:**
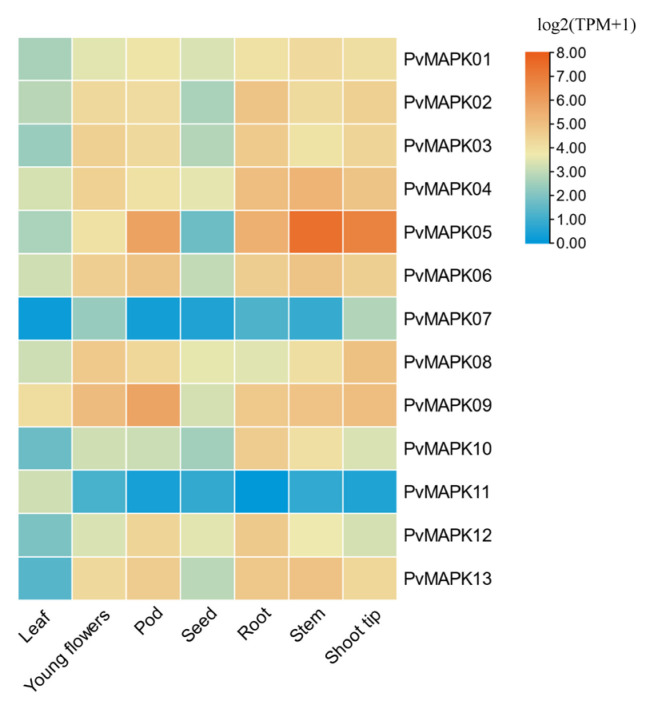
Expression of *PvMAPK* genes in seven tissues of *P. vulgaris.*

To further investigate the potential functions of the *PvMAPK* genes in growth and development, we downloaded transcriptome data for leaves, flowers, pods, seeds, roots, stems, and stem tips of *P. vulgaris* from the Legume Information System (https://www.legumeinfo.org/ accessed on 26 November 2024 [21]) database and used these data to analyze *PvMAPK* gene expression (Figure 9). The *PvMAPK* genes were expressed in all tissues examined, although the expression of individual *PvMAPKs* differed among plant parts. *PvMAPK05* showed particularly high expression in stems, stem tips, and (to a lesser extent) pods. By contrast, expression of *PvMAPK07* and *PvMAPK11* was relatively low across all tissues. In general, *PvMAPK* expression was lower in leaves and seeds and higher in stems, stem tips, and pods, suggesting that *PvMAPKs* may have a particularly important role in the regulation of stem and pod development.

### 2.6. Expression of PvMAPKs in Seven Oil Bean Varieties Under Anthracnose Stress

**Figure 10:**
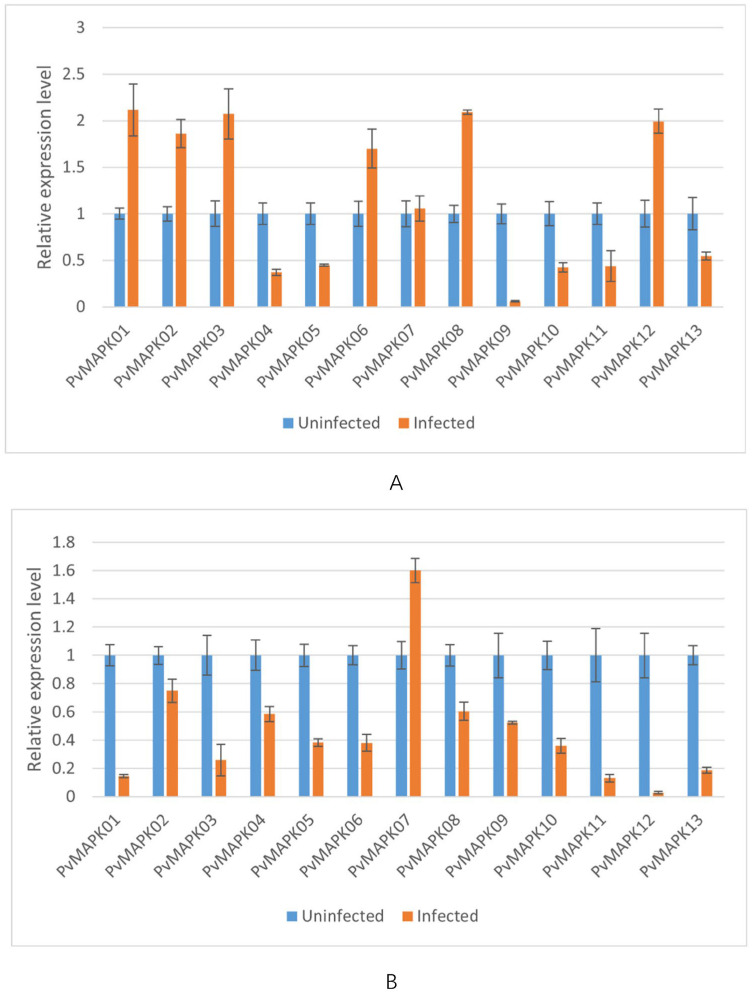
qRT-PCR analysis of *PvMAPK* gene expression in field-grown *P. vulgaris* ‘HuangJinGou’ plants with or without anthracnose infection. Relative expression in leaves (**A**) and stems (**B**) is shown, and values for anthracnose-infected plants are normalized to those of uninfected plants, which were set to 1.

**Figure 11:**
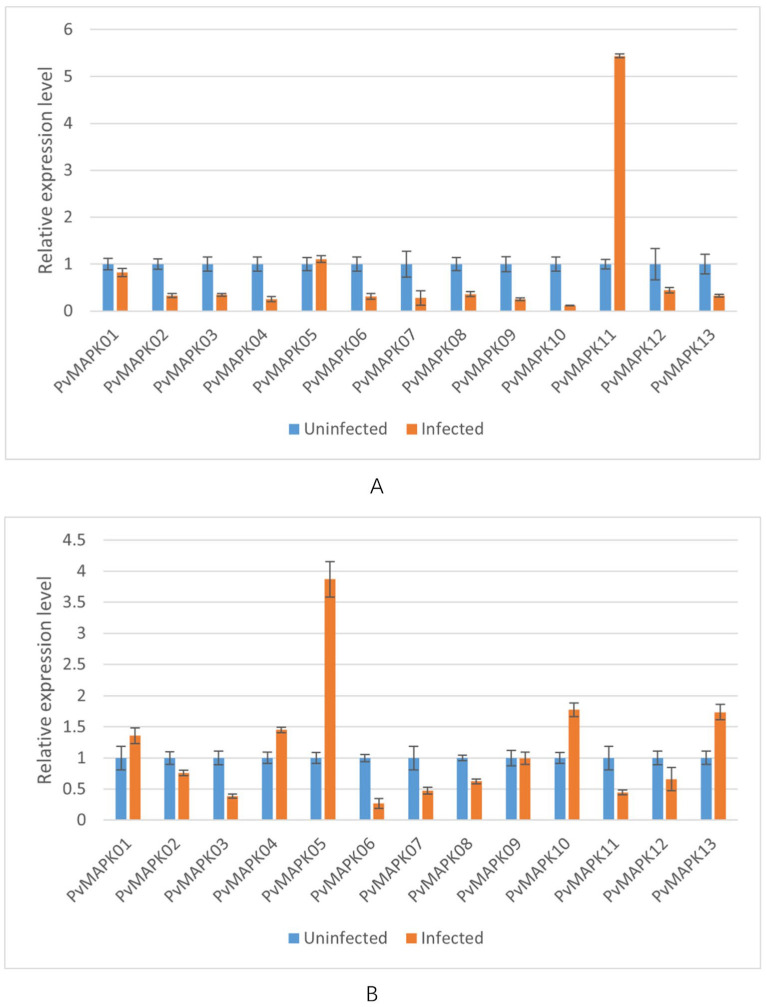
qRT-PCR analysis of *PvMAPK* gene expression in field-grown *P. vulgaris* ‘BaiYunFeng’ plants with or without anthracnose infection. Relative expression in leaves (**A**) and stems (**B**) is shown, and values for anthracnose-infected plants are normalized to those of uninfected plants, which were set to 1.

**Figure 12:**
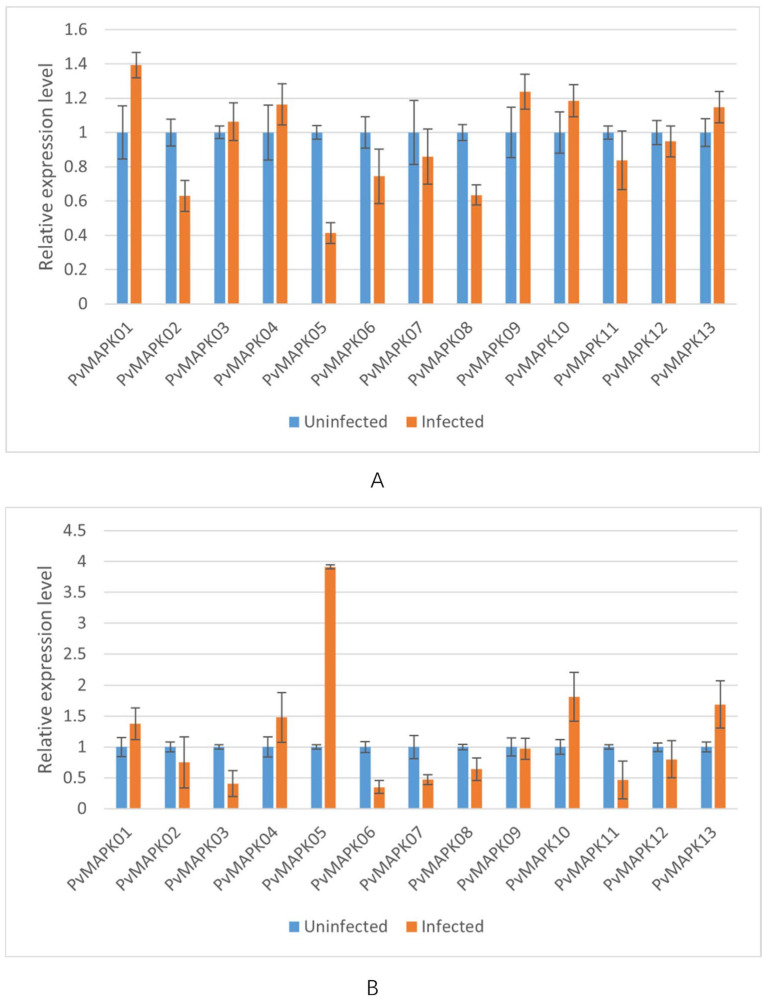
qRT-PCR analysis of *PvMAPK* gene expression in field-grown *P. vulgaris* ‘Jifeng Oil Bean’ plants with and without anthracnose infection. Relative expression in leaves (**A**) and stems (**B**) is shown, and values for anthracnose-infected plants are normalized to those of uninfected plants, which were set to 1.

**Figure 13:**
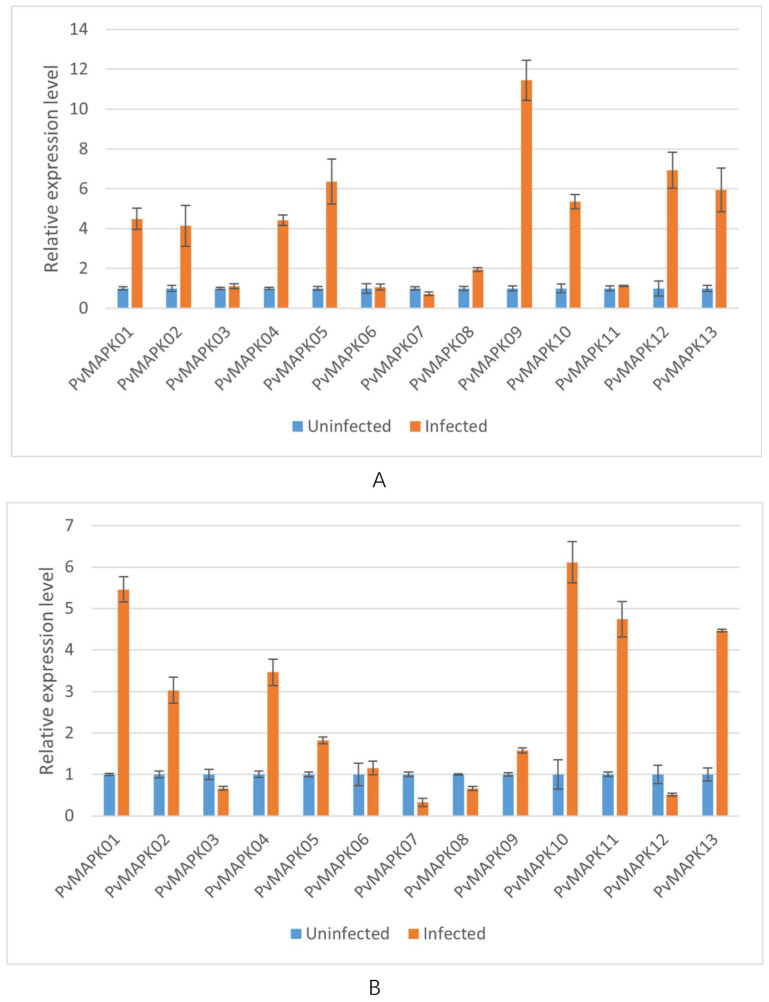
qRT-PCR analysis of *PvMAPK* gene expression in field-grown *P. vulgaris* ‘Giant Oil Bean’ plants with or without anthracnose infection. Relative expression in leaves (**A**) and stems (**B**) is shown, and values for anthracnose-infected plants are normalized to those of uninfected plants, which were set to 1.

**Figure 14:**
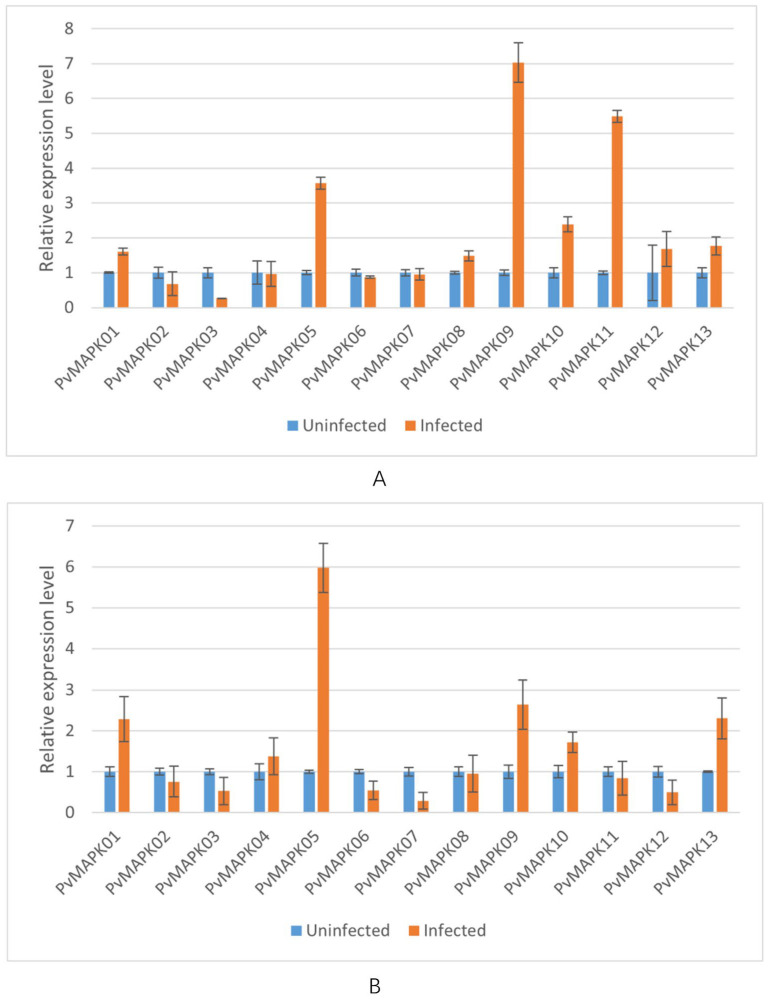
qRT-PCR analysis of *PvMAPK* gene expression in field-grown *P. vulgaris* ‘YiKeSong’ plants with or without anthracnose infection. Relative expression in leaves (**A**) and stems (**B**) is shown, and values for anthracnose-infected plants are normalized to those of uninfected plants, which were set to 1.

**Figure 15:**
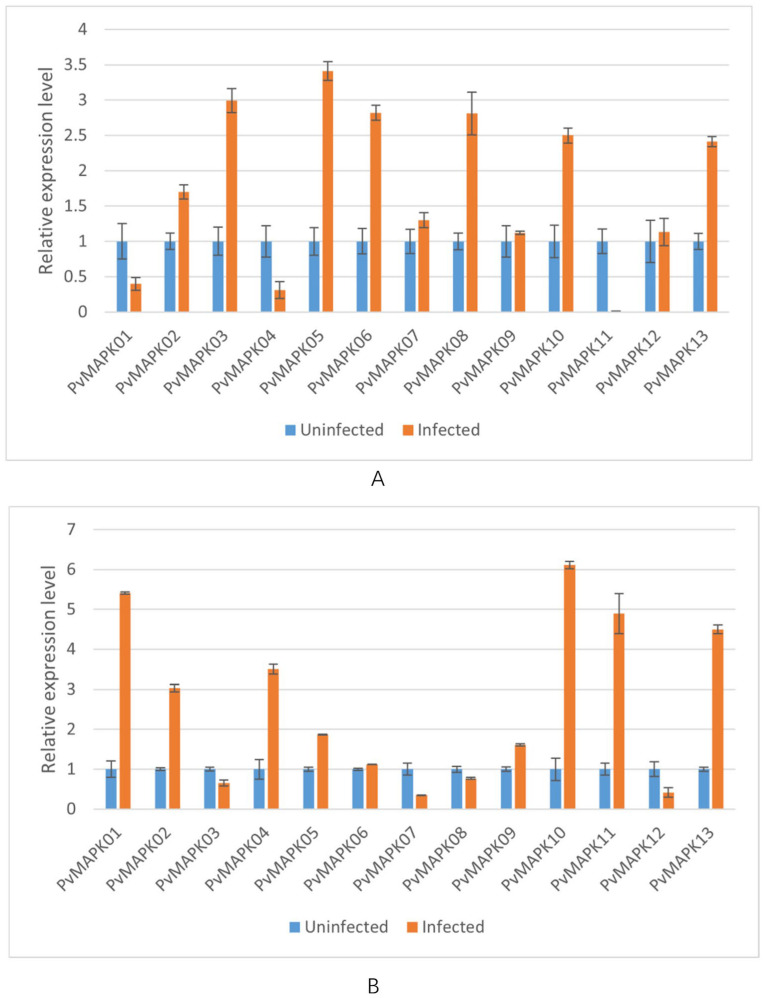
qRT-PCR analysis of *PvMAPK* gene expression in field-grown *P. vulgaris* ‘Wuchang Big Oil Bean’ plants with or without anthracnose infection. Relative expression in leaves (**A**) and stems (**B**) is shown, and values for anthracnose-infected plants are normalized of those in uninfected plants, which were set to 1.

**Figure 16:**
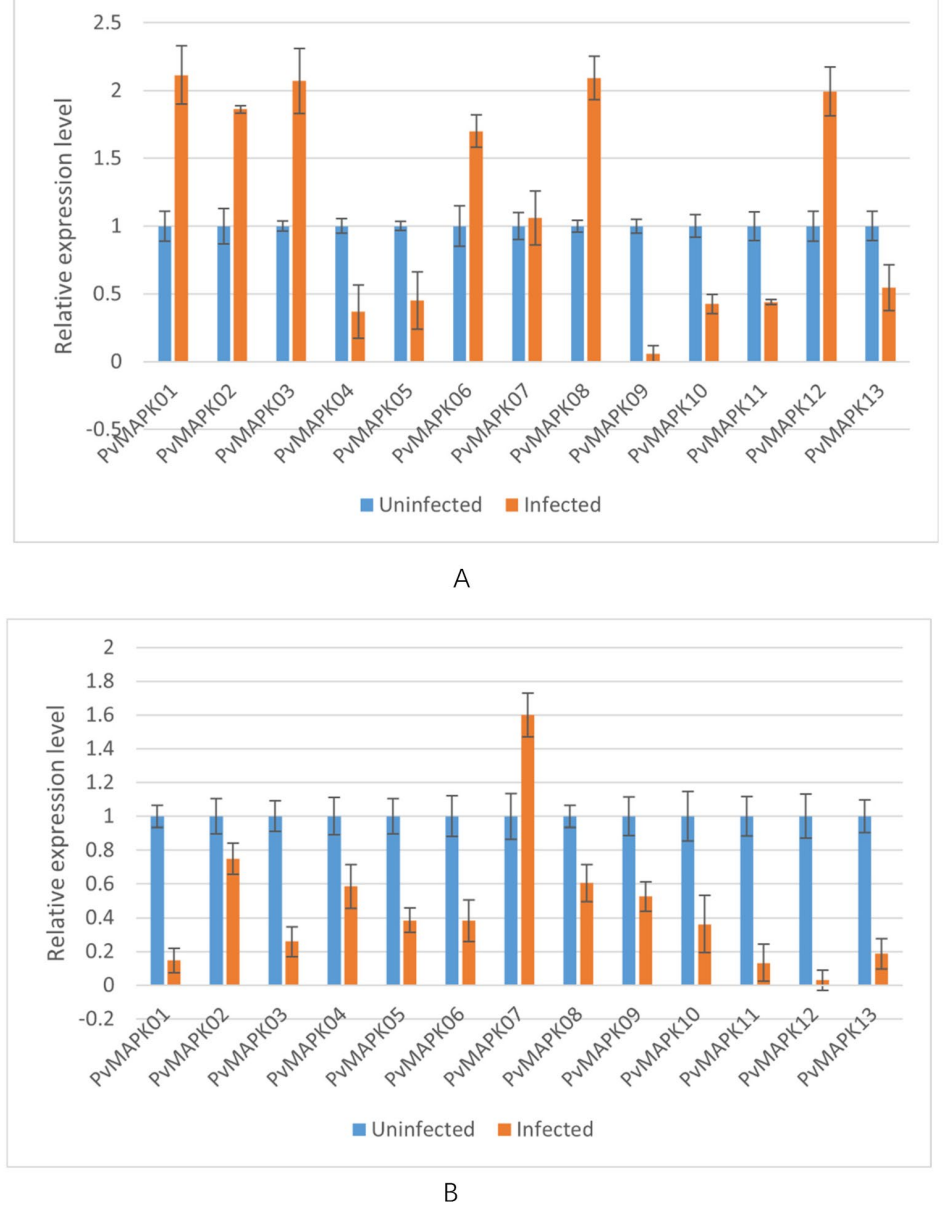
qRT-PCR analysis of *PvMAPK* gene expression in field-grown *P. vulgaris* ‘P9 oil bean’ plants with or without anthracnose infection. Relative expression in leaves (**A**) and stems (**B**) is shown, and values for anthracnose-infected plants are normalized to those of uninfected plants, which were set to 1.

We next examined expression of the *PvMAPKs* in leaf and stem tissue from seven varieties of field-grown oil bean plants, with and without natural anthracnose infection (Figure 10, Figure 11, Figure 12, Figure 13, Figure 14, Figure 15 and Figure 16).

In the variety ‘HuangJinGou’, seven *PvMAPK* genes (*PvMAPK01/02/03/06/07/08/12*) were upregulated 1.05- to 2.11-fold in infected leaves compared with uninfected leaves, with *PvMAPK01* showing the greatest upregulation. The remainder of the *PvMAPK*s were downregulated to various extents in infected leaves, with PvMAPK09 exhibiting the greatest downregulation. In the stems, only *PvMAPK07* was upregulated (1.60-fold) upon infection, and the remaining *PvMAPK*s were downregulated.

In infected leaves of ‘Baiyunfeng’, *PvMAPK05* and *PvMAPK11* were upregulated by 1.10- and 5.61-fold, respectively, and the remaining *PvMAPKs* were downregulated (Figure 11). In infected stems, expression of *PvMAPK01*, *PvMAPK04*, *PvMAPK05*, *PvMAPK10*, and *PvMAPK13* was upregulated 1.35- to 3.86-fold, with *PvMAPK05* showing the greatest upregulation; expression of the remaining genes was downregulated.

In infected leaves of ‘Jifeng’, most *PvMAPK* genes showed relatively little change in expression. Their expression levels ranged from 0.41 to 1.39 times those of uninfected leaves, with *PvMAPK05* showing the greatest downregulation and *PvMAPK01* showing the greatest upregulation. By contrast, *PvMAPK05* was upregulated 3.91 fold in infected ‘Jifeng’ stems, and *PvMAPK01*/*04*/*10*/*13* were also upregulated. *PvMAPK06* showed the greatest downregulation in infected stems (0.35-fold relative to uninfected stems).

Multiple *PvMAPK* genes were upregulated in infected leaves and stems of ‘Giant Oil Bean’ (Figure 13). The most substantial upregulation was observed for *PvMAPK09* in leaves (11.44-fold) and *PvMAPK10* in stems (6.11-fold). Other genes showed various degrees of up- and downregulation.

Seven genes (*PvMAPK01/05/09/10/11/12/13*) were upregulated in infected leaves of ‘YiKeSong’, with *PvMAPK09* showing the greatest upregulation (7.02-fold relative to uninfected leaves) (Figure 14). *PvMAPK11* was also strongly upregulated (5.48-fold). In the stems, six *PvMAPKs* were upregulated, with *PvMAPK05* expression showing the greatest increase (5.97 fold).

In infected leaves of ‘Wuchang Big Oil Bean’, all *PvMAPKs* except *PvMAPK01*, *PvMAPK04*, and *PvMAPK11* were upregulated (Figure 15). *PvMAPK05* exhibited the greatest upregulation (3.41-fold). In infected stems, all *PvMAPKs* except *PvMAPK03*/*07*/*08/12* were upregulated, with *PvMAPK10* showing the greatest upregulation (6.11-fold) and *PvMAPK07* the greatest downregulation (0.35-fold).

Seven *PvMAPKs* were upregulated in infected leaves of the variety ‘P9’ (*PvMAPK01/02/03/06/07/08/12*), most by approximately two-fold compared with uninfected leaves. By contrast, only *PvMAPK07* was upregulated (1.60-fold) in infected stems. The remaining twelve genes were downregulated to various degrees, with *PvMAPK12* showing the most pronounced decrease (0.02-fold).

In summary, the modulation of expression levels of MAPK gene family members within the leaf and stem tissues of seven *P. vulgaris* varieties is associated with the stress imposed by anthracnose. Notably, the expression patterns of *PvMAPK05*, *PvMAPK07*, *PvMAPK09*, and *PvMAPK11* across different varieties exhibit notable similarities, suggesting their potential role in the *P. vulgaris*’s response to anthracnose stress. However, the precise mechanisms underlying their response remain to be clarified. It is essential to conduct further research to evaluate their potential in disease resistance.

## 3. Discussion

The MAPK cascade plays pivotal roles in the regulation of plant growth and development, as well as biotic and abiotic stress responses. Environmental signals are transmitted from receptor proteins to MAPKKKs, MAPKKs, and MAPKs through sequential phosphorylation, and MAPKs regulate growth, development, and stress by phosphorylating downstream substrates or promoting the expression of related genes [22,23]. In the existing literature, known downstream signal sensors include NPR1 regulator, the 14-3-3 proteins GRF6 and GRF8, and CcSte12 [22,23].

In recent years, NPR1, as a regulatory protein, has been found to be required in the development of inducible resistance induced by pathogen infection. NPR1 enters the nucleus and activates PR1 gene expression via the TGA transcription factor. Riboflavin treatment induced PR1 expression in WT plants after challenge. However, riboflavin preconditioning did not promote increased PR1 transcription in npr1 mutants. The relationship between MAPKs and NPR1 after Pst DC3000 inoculation was further studied. Inoculation with Pst DC3000 promoted the expression of NPR1 protein in WT plants, while NPR1 expression was almost suppressed in mpk3 and mpk6 mutants. These data suggest that riboflavin induces defense activation through NPR1-dependent signaling pathways in response to Pst DC3000, and that riboflavin-induced MAPKs signaling modules may work upstream of NPR1 regulators [24].

It was found that the Arabidopsis 14-3-3 proteins GRF6 and GRF8 play a key role in the regulation of PTI. These two isomers interact with RLCKs and induce defense gene expression, callose deposition, and resistance to two bacteria (Pseudomonas syringae pv). Further characterization showed that these two isomers were required for pattern-triggered MAP kinase activation, but not for other early signaling events such as reactive oxygen species bursts (Figure 3) and phosphorylation of BIK1 and RGS1 (Figure 6). We found that GRF6 directly interacts with the C-terminus of MAPKKK5 to promote access to it by immune-activated PBL19 (Figure 6, revealing a unique mechanism by which patterns trigger MAP kinase activation and immunity. Through phosphorylated proteomic analysis and yeast two-hybrid experiments, the researchers demonstrated that CcPmk1 can phosphorylate and interact with the downstream homeobox transcription factor CcSte12. Phosphorylated proteomic analysis of the CcPmk1 deletion mutant and the wild type strain showed that the phosphorylation level of CcSte12 was significantly reduced in the CcPmk1 deletion mutant compared to the wild type strain. In addition, the abundance of peptides containing phosphorylated residues CcSte12Ser405, CcSte12Ser487, and CcSte12Ser545 in CcPmk1 deletion mutants was significantly reduced compared to the wild type strain. In addition, the phosphorylated residue CcSte12Ser405 contains the MAPK S/T-P phosphorylation motif. Deletion of CcPmk1 significantly reduces but does not eliminate phosphorylation of CcSte12 (Yu et al. 2022), suggesting that CcSte12 is also phosphorylated by kinase proteins other than CcPmk1, and that CcPmk1 has some additional downstream transcription factors [25].

Here, we identified 13 members of the *MAPK* gene family in *P. vulgaris* through homology searches and confirmed their identity on the basis of conserved domains in their predicted proteins. This work complements previous studies that have identified *MAPK* genes in *Arabidopsis* (20 genes) [9], *B. distachyon* (16) [10], *Z. mays* (19) [11], *H. vulgare* (20) [12], *T. aestivum* (54) [14], and *O. sativa* (17).

All PvMAPK proteins contained the conserved T(D/E)YXXTRWYRAPEL motif, and phylogenetic analysis placed the PvMAPKs into four previously described subgroups [6]: six PvMAPK proteins with the TEY motif in groups A–C and seven PvMAPK proteins with the TDY motif in group D. The larger number of proteins in group D is consistent with other findings in dicots. The TDY motif is located in Motif 4, and the TEY motif is situated in Motif 7. This is consistent with the analysis results of other plants such as Arabidopsis, barley, sorghum, Brachypodium, and chickpea [10,12,26,27,28].

The number and distribution of exons and introns in the gene structure of *PvMAPKs* demonstrate a notable degree of regularity across subfamilies. Groups A and B consist of six exons, while group C has just two exons. Group d stands out with a larger number of exons, specifically between 9 and 11. This pattern of gene structure is mirrored in other plants, characterized by a high degree of conservation within each group and significant divergence between groups [29].

The duplication of individual genes, chromosome segments, or whole genomes is the main driving force for genome formation and evolution [30]. There are five syntenic *MAPK* gene pairs on the *P. vulgaris* chromosomes. *PvMAPK11* and *PvMAPK12* are both located on chromosome 2 but appear to represent segmental rather than tandem duplicates. The remainder of the pairs are found on different chromosomes. This pattern suggests that whole-genome duplications and chromosome segment translocations may have played important roles in the expansion of the *MAPK* gene family of *P. vulgaris* [31]. Collinearity analyses of P. vulgaris with soybean and Arabidopsis revealed that 77% of the *PvMAPK* genes were collinear with their homologs in the Arabidopsis genome. This is also consistent with the research findings in *Nelumbo nucifera* by Gaerth [32], but differs from the collinearity studies between *Setaria italica* MAPK genes and Arabidopsis MAPK genes [32]. This may be due to divergence of the MAPK gene family during the evolutionary process between monocots and dicots. All were collinear with their homologs in the soybean genome. This finding suggests a close evolutionary relationship between the MAPK genes of P. vulgaris and those of Arabidopsis and soybean.

Similar studies on MAPK genes in other legumes showed that the Glyma.18G268800 gene in soybean may participate in resistance to Phytophthora root rot through the MAPK signaling pathway [32]. In peas, the expression of PsMAPK3 gene is associated with resistance to A. pisense [33]. These studies further demonstrated the extensive role of MAPK signaling pathway in plant disease resistance and also provided better background and an important basis for this study. In summary, MAPK cascades are an interesting target for breeding disease-resistant varieties because they play a key regulatory role in the plant immune response. Through in-depth research and utilization of MAPK signaling pathways, new strategies and methods can be provided for improving plant disease resistance.

Analysis of the 2 kb upstream sequences of the promoter regions of the Phaseolus vulgaris Mitogen-Activated Protein Kinase (MAPK) gene family members can provide valuable insights into their potential gene functions. This study identified 14 distinct types of regulatory elements within the promoter regions of the PvMAPKs encoding proteins. Among these elements, there are biotic stress regulatory cis-elements, including jasmonic acid response cis-regulatory elements, salicylic acid response defense cis-acting elements, and defense and stress response cis-acting elements, which are crucial for plant stress tolerance regulation. Jasmonic acid response cis-regulatory elements and salicylic acid response defense cis-acting elements are predominantly found in stress-related genes, highlighting their role in modulating plant responses to various stresses [34]. Additionally, the abscisic acid pathway is instrumental in activating defense genes during plant responses to both abiotic and biotic stresses [35]. SlMAPK3 in tomato is involved in the antiviral response to yellow leaf curl virus (TYLCV). VIGS silencing of SlMAPK3 can increase viral infection, decrease the expression of defense-related genes and the plant’s tolerance to TYLCV. Overexpression of SlMAPK3 enhances the expression of defense-related genes and increases tolerance to TYLCV [36]. In rice, the OSMKK4-OSMPK3/OsMPK6 cascade is activated by MAMP signaling, inducing immune responses such as defense-related gene expression, antimicrobial compound synthesis, and cell death, but does not produce ROS. OsMPK6 plays a major role in the regulation of diterpenoid plant protection hormone synthesis and plant cell death induced by chitin, but has no effect on the synthesis of phenylpropyl compounds [5]. In barley, HvMPK4 is a negative regulator of basal resistance, acting upstream of HvWRKY1. Hvmkk1-hvmpk4 kinase phosphorylates HvWRKY1 and regulates the immunity of barley against barley powdery fungi (Bgh) [37]. In Arabidopsis, MYB44 collaborates with the MPK3/6 cascade and EIN2 to regulate PTI development. PAMPs induce the expression of MYB44, MPK3, and MPK6; MYB44 activates the expression of MPK3 and MPK6; and phosphorylated MPK3 and MPK6 in turn enable MYB44 to activate MPK3, MPK6, EIN2, and downstream defense responses [38].

Within the common bean MAPK gene family, 11 members have been found to contain both jasmonic acid response cis-regulatory elements and salicylic acid response defense cis-acting elements. This suggests a significant role for the common bean MAPK gene family in the regulation of plant stress responses. Furthermore, the presence of a substantial number of light response elements in PvMAPKs indicates their potential importance in the modulation of light signals in common beans, a finding that aligns with research conducted in lotus [39] and wheat [40]. In cotton studies, it was found that the up-regulated expression of GHMAPK-related cis-regulatory elements significantly enhanced tolerance to drought and salt stress [41]. The researchers identified the StMAPK1 gene in potato, which maintains a high expression level under high temperature stress and reduces the ion permeability of cell membrane by increasing the content of proline and reducing the content of malondialdehyde, so as to ensure the stability of the cell membrane, thereby improving the heat resistance of the potato [34]. Arabidopsis thaliana overexpressing zmmkk1 promotes stomatal closure or effective removal of excess reactive oxygen species in an ABA-dependent manner, enhancing tolerance to salt and drought stress [42]. Overexpression of the maize MAPKK gene ZmMPK7 in transgenic tobacco can enhance the protection of the acne defense system against ROS-mediated injury under osmotic stress [43]. The researchers identified an osmapkkk43 mutant in rice which imparts broad-spectrum resistance to Mixanthomonas pv [44].

Anthracnose is an important fungal disease of the oil bean, with a considerable impact on both yield and quality. A substantial body of research has demonstrated that *MAPK* genes play pivotal roles in the regulation of pathogen and virus resistance across a diverse range of plant species. In citrus, *CsMAPK1* is induced by *P. ulcerans* and plays a role in regulating bacterial resistance [45]. In tobacco, *MAPK* expression can be activated by a variety of biotic stresses and defense hormones [46,47]. In Arabidopsis, MAPK3 and MAPK6 are involved in riboflavin-induced resistance to the tomato pathogenic variant of Pseudomonas syringae (Pst DC3000), and MPK15 regulates resistance to powdery mildew [48]. HvMPK4 in barley is a negative regulator of basal resistance in barley and inhibits immunity to barley powdery mildew fungi [49]. The MKP1–MAPK cascade positively regulates vascular defenses by activating lignin biosynthesis and has a positive effect on nonhost resistance (NHR) in monocotyledonous rice and dicotyledonous Arabidopsis thaliana and on host resistance to vascular pathogens [50]. In the soybean, the Glyma.18G268800 gene was found to be involved in resistance to Phytophthora root rot through the MAPK signaling pathway [51]. In peas, expression of the PsMAPK3 gene is associated with resistance to A. pisense [52].

Expression of PVMAPK05 is high in the stem and pod, which may be the first sites invaded or vulnerable to the pathogen under biological stress (anthrax infection). The high expression of PvMAPK05 in these tissues may enable the plant to rapidly sense the invasion signals of pathogenic bacteria. Since the MAPK cascade plays a key role in plant signal transduction, PvMAPK05 may act as an early signal sensing and conduction molecule, transmitting signals of pathogen invasion to downstream defense-related genes or signaling pathways [6]. For example, when anthrax infects the stem, PvMAPK05 may be activated through its conserved domain (such as containing TEY or TDY phosphorylation sites) and then phosphorylate downstream substrates (such as transcription factors or other kinases), thus initiating the expression of defense-related genes, such as genes involved in plant cell wall strengthening, synthesis of antimicrobial substances, and so on.

The tissue structures and physiological functions of the stem, tip, and pod are different, and their defense responses to biological stress may also be specific. The high expression of PvMAPK05 in these tissues may help regulate tissue-specific defense responses. In stems, it may be involved in regulating lignin synthesis to enhance the mechanical strength of stems and present a physical barrier to pathogens. In the pod, it may be related to regulating the closure mechanism of the pod or the accumulation of antibacterial substances in the pod to protect the seeds from pathogens. In addition, the high expression of PvMAPK05 may work synergistically with other defense-related genes in these tissues to form a multilayered defense system. For example, it may interact with PR genes (disease course-related genes) that are involved in the plant’s immune response to regulate the plant’s resistance to anthrax.

In this study, the relative expression levels of *PvMAPK* genes in anthracnose-infected and uninfected tissues varied among different oil bean varieties. The relative expression levels of *PvMAPK05*, *PvMAPK07*, *PvMAPK09*, and *PvMAPK11* exhibited particularly marked changes in response to anthracnose infection across multiple varieties, suggesting that they may play a role in the anthracnose response of *P. vulgaris*. However, the precise response mechanism remains to be clarified, and further research is necessary to assess their potential roles in disease resistance.

## 4. Materials and Methods

### 4.1. MAPK Gene Identification and Protein Characterization

Ation Resource (TAIR) 10 database (https://www.arabidopsis.org/, accessed on 26 November 2024) and rice MAPK protein sequences from the Phytozome database (https://phytozome-next.jgi.doe.gov/, accessed on 26 November 2024). These sequences were used as BLASTP queries to identify candidate MAPK genes in *P. vulgaris* by searching against the *P. vulgaris* genome protein sequences (taxid:3885) at NCBI. These candidate sequences were identified by searching the NCBI (https://www.ncbi.nlm.nih.gov/Structure, accessed on 26 November 2024) “CD-search” Accession: cd07858 and cd07859, SMART (https://smart.embl.de/, accessed on 26 November 2024), and PFAM databases (http://pfam.xfam.org/, accessed on 26 November 2024) (PFAM ID:PF00069, PF07714) for structural domain validation again to ensure that they have conserved structural domains specific to MAPK. Candidates with missing or incomplete MAPK structural domains were removed, and the remaining genes were identified as P. vulgaris MAPK genes. We examined the physiochemical properties of their encoded proteins using ExPASy-PROSITE (https://prosite.ExPASy.org/, accessed on 26 November 2024), predicted the protein subcellular localizations with WOLF PSORT (https://wolfpsort.hgc.jp), and searched for signal peptides using SignalP-6.0 (https://services.healthtech.dtu.dk/services/SignalP-6.0/, accessed on 26 November 2024). To visualize the positions of the *PvMAPKs* on chromosomes, we downloaded the *P. vulgaris* genome sequences and gene annotations from Phytozome (https://phytozome-next.jgi.doe.gov/, accessed on 26 November 2024) and used them as input for the ‘Visualize Gene Structure (from GTF/GFF3 File)’ module of TBtools (Version 1.108).

### 4.2. Analyses of Protein Phylogeny and Gene Collinearity

Firstly, we conducted a multiple sequence alignment of the MAPK gene family sequences from the cauliflower bean using MEGA11.0 software. The alignment was performed with the following parameters: Gap Open set to −2.90, Gap Extend to 0.00, and Hydrophobicity Multiplier to 1.20. We limited the number of iterations to 16. For the initial iterations (1.2), we employed the UPGMA clustering method, which was also used for subsequent iterations. Additionally, we set the minimum diagonal length (Lambda) to 24 to ensure the alignment’s precision and consistency. Then we constructed a neighbor-joining phylogenetic tree of MAPK protein sequences from *P. vulgaris* (13 sequences), *Arabidopsis* (20), and *O. sativa* (17) using MEGA 11.0 software with the Jones–Taylor–Thornton (Tl) model substitution model, 1000 bootstrap replicates, and pairwise deletions. A phylogenetic tree for the 13 *P. vulgaris* proteins alone was constructed using the same method.

Genome sequence files and gene annotation files for P. vulgaris, Arabidopsis, rice, and soybean were downloaded from the Phytozome database (https://phytozome-next.jgi.doe.gov/, accessed on 26 November 2024). Intraspecific and interspecific collinearity of MAPK genes was analyzed using the ‘One Step McscanX’ and ‘Dual Synteny Plot’ functions in TBtools. Intragenomic Ka/Ks values for collinear gene pairs were calculated using the ‘Simple Ka/Ks Calculator (NG)’ in TBtools.

### 4.3. Protein Motifs and Gene Structures of the PvMAPKs

Motifs were predicted in the PvMAPK proteins using MEME tools (https://meme-suite.org/meme/tools/meme, accessed on 26 November 2024) with the number of motifs set to 15 and the remaining parameters set to default values. The ‘Gene Structure View (Advanced)’ function of TBtools was used to visualize intron–exon structures, protein motifs, and conserved domains, together with the PvMAPK phylogenetic tree.

### 4.4. Identification of Cis-Acting Promoter Elements

Promoter sequences of the *P. vulgaris* MAPK genes (2000 bp upstream of the start codon) were extracted using TBtools and submitted to the Plant CARE website (http://bioinformatics.psb.ugent.be/webtools/plantcare/html/, accessed on 26 November 2024) for prediction of *cis*-acting promoter elements. The results were visualized using the ‘Basic Biosequence View’ function of TBtools.

### 4.5. Expression of PvMAPKs in Different Tissues and in Response to Anthracnose Infection

Transcriptome data (in transcripts per million, TPM) for seven *P. vulgaris* tissues were downloaded from the Legume Information System database (https://www.legumeinfo.org, accessed on 26 November 2024). The corresponding log2(TPM + 1) values were calculated and visualized using the ‘Heatmap’ function in TBtools.

### 4.6. Expression of PvMAPKs in Seven Oil Bean Varieties Under Anthracnose Stress

To investigate the effect of anthracnose infection on PvMAPK expression, leaf and stem tissues were sampled from seven field-grown P. vulgaris varieties (‘HuangJinGou’, ‘BaiYunFeng’, ‘Jifeng Oil Bean’, ‘Giant Oil Bean’, ‘YiKeSong’, ‘Wuchang Big Oil Bean’, and ‘P9 oil bean’), with and without naturally occurring anthracnose infection. The plant materials were grown at the Vegetable Teaching and Research Station of Jilin Agricultural University in Changchun, Jilin, China (geocoordinates: longitude 125.406455, latitude 43.816967). Samples were collected at the peak pod-setting stage, with five replicates obtained for each treatment. Following collection, the samples were consolidated, portioned into individual packages, and swiftly frozen in liquid nitrogen for storage, ready for the subsequent extraction of total RNA.

Total RNA was extracted from leaf and stem tissues using the TransZol Up plus RNA kit (TRAN, ER501) and reverse transcribed using a TRAN reverse transcription kit (AU301). qRT-PCR was performed using TB Green Premix Ex Taq II (Tli RNaseH Plus) (TakaRa, RR820A, Tokyo, Japan) on a qTower^3^ (230 V) (Jena Analytical Instruments (Beijing) Co., Beijing, China) instrument. The 20 μL reaction mixture consisted of 10 μL TB Green Premix Ex Taq II, 0.8 μL each forward and reverse primers, 1 μL cDNA template, and 7.4 μL sterilized double-distilled water. The reaction protocol consisted of an initial denaturation at 95 °C for 30 s, followed by 40 cycles of denaturation at 95 °C for 5 s and annealing and extension at 60 °C for 30 s, with fluorescence signals collected at each cycle. Each reaction was performed in triplicate, and the relative expression levels of the MAPK genes were calculated using the 2^−ΔΔCt^ method. Fluorescent quantitative primers were designed using Premier 5.0 software (Additional file 12, Table 3) and synthesized by Shenggong Bioengineering (Shanghai, China). The *P. vulgaris* actin gene was used as an endogenous control.

## 5. Conclusions

This study conducted a thorough investigation of the MAPK family in Phaseolus vulgaris, identifying a total of 13 PvMAPKs, which were classified into four subfamilies. In-depth analyses of their physicochemical properties, chromosomal locations, phylogenetic relationships, gene structures, conserved motifs, cis-acting elements, and collinearity shed light on their potential roles in evolution and function. Subsequently, plant materials from seven P. vulgaris varieties were employed to assess the relative expression levels of PvMAPKs under conditions of anthracnose stress. The findings revealed a correlation between upregulation and downregulation of the 13 PvMAPKs’ expression levels and anthracnose stress. Among the identified genes, PvMAPK05, PvMAPK07, PvMAPK09, and PvMAPK11 exhibited notably variable expression levels across several distinct cultivars, leading to the hypothesis that these PvMAPKs may play a significant role in the response to anthracnose stress. This variation in expression patterns suggests a potential regulatory function of these genes in the stress response mechanism of Phaseolus vulgaris. Furthermore, the insights gained from this study lay the groundwork for future studies aimed at the functional validation of these genes.

## Figures and Tables

**Table 1:** Basic information on PvMAPK family members in *P. vulgaris*.

Gene Name	Gene ID	Number of Amino Acids (aa)	Molecular Weight (kD)	Isoelectric Point (pI)	Subcellular Location	Signal Peptide
*PvMAPK01*	PAC:27140243	374	42.86	6.14	cyto	NO
*PvMAPK02*	PAC:27142484	395	45.06	5.57	cyto	NO
*PvMAPK03*	PAC:27144563	570	64.7	8.94	cyto	NO
*PvMAPK04*	PAC:27150399	607	68.97	9.21	nucl	NO
*PvMAPK05*	PAC:27151651	372	42.65	5.65	cyto	NO
*PvMAPK06*	PAC:27155619	614	69.98	9.15	nucl	NO
*PvMAPK07*	PAC:27158371	506	57.75	6.17	cyto	NO
*PvMAPK08*	PAC:27165246	603	68.41	8.97	cyto	NO
*PvMAPK09*	PAC:27166653	564	64.11	8.82	cyto	NO
*PvMAPK10*	PAC:27167601	369	42.48	8.28	cyto	NO
*PvMAPK11*	PAC:27170661	382	43.66	6.4	cyto	NO
*PvMAPK12*	PAC:27170962	373	42.9	5.83	cyto	NO
*PvMAPK13*	PAC:27171377	583	66.11	7.09	cyto	NO

**Table 2:** Ka/Ks values of PvMAPK gene pairs.

Gene 1	Gene 2	Non-Synonymous (Ka)	Synonymous (Ks)	Ka/Ks
*PvMAPK12*	*PvMAPK11*	0.083641093	0.756503346	0.110562754
*PvMAPK11*	*PvMAPK01*	0.123021052	1.646005777	0.074739137
*PvMAPK12*	*PvMAPK01*	0.130405085	1.952985631	0.066772168
*PvMAPK03*	*PvMAPK09*	0.052767243	0.608554437	0.086709159
*PvMAPK08*	*PvMAPK06*	0.092142646	0.6611902	0.139358759

**Table 3:** Primer sequences for qRT-PCR of MAPK genes in *P. vulgaris*.

Gene	Forward Sequence (5′-3′)	Reverse Sequence (5′-3′)
*Pv-Actin*	GAAGTTCTCTTCCAACCATCC	TTTCCTTGCTCATTCTGTCCG
*PvMAPK01*	TGGTGGACGCTACATTCAGT	TGCATGATCCATGTGCCGAA
*PvMAPK02*	GTTGCGTCTGCTTATGGAGC	GTGCCAGGGCATCTTCAACA
*PvMAPK03*	GACGGAGAAGAGCCAACAGG	AAAGGAGGCCTGGTCATTGG
*PvMAPK04*	CCAGAACTGTGTGGCTCCTT	TGCAGTAGACGAAGTGCCAA
*PvMAPK05*	CTTCTTGGTACCCCAACCGA	AGTGCTTCTTCAACTGTAATTCTTT
*PvMAPK06*	ACAGACAATCCTGCCCCAAG	TCTGTTGCTGTTGCTGACCT
*PvMAPK07*	ACCACCGGAGAAATCGATGC	AGGGAAGCAACCTTCTGTGT
*PvMAPK08*	AAACCGACCAACTGCTGAGG	TAAGCTGGGGGAACAGGTTG
*PvMAPK09*	AGACCCCCTAGCTCTTCGTT	ATCCGGCGTCTCTCAAACTC
*PvMAPK10*	AAGTCCTCGCAGCCACTTTC	GTTCGTGCAAGCCCAAAGTC
*PvMAPK11*	TTCCACAGTACCGGAAGCAA	CTGGGACAGACTGGCTCATC
*PvMAPK12*	GACCTCCCAGAAAGGATGCC	CACCGGGTGACCACATACTC
*PvMAPK13*	CGCGCAGTTGTCTTCCATTC	GTGAGCATCAACGGCAGAAC

## Data Availability

All data generated or analyzed during this study are included in this published article or its additional files. The datasets used and/or analyzed during the current study are available from the authors on reasonable request (Huiling Liu, liuhuiling@mails.jlau.edu.cn).
